# Retraction: An Often Easily Missed Injury in the Presence of Orthopaedic Trauma: A Case Series of Derived Injury

**DOI:** 10.1111/os.12972

**Published:** 2021-02-17

Retraction: Deng, X.‐t., Wang, Z.‐z., Zhu, J., Tan, Z.‐c., Wang, Y.‐c., Zhu, Y.‐b., Chen, W. and Zhang, Y.‐z. (2020), An Often Easily Missed Injury in the Presence of Orthopaedic Trauma: A Case Series of Derived Injury. Orthop Surg, 12: 337‐342. https://doi.org/10.1111/os.12606

The above article^1^, published online on 20 January 2020 in Wiley Online Library (wileyonlinelibrary.com), has been retracted by agreement between the authors, the journal Editor in Chief and John Wiley & Sons Ltd. The retraction has been agreed due to unattributed overlap of figures with two articles previously published in Archives of Orthopaedic and Trauma Surgery “Superior gluteal artery injury presenting as delayed onset shock” by Zhang Q, Chen W, Smith WR, Pan J, Liu H, Zhang Y. 2010, 130(2):251‐256 and Acta Orthopaedica et Traumatologica Turcica “Occult external iliac vein injury after anterior dislocation of the sacroiliac joint in adult patient” by Liu Y, Wang J, Zhang Y. 2017, 51(2):169‐171.
